# Adverse effects of iron deficiency anemia on pregnancy outcome and offspring development and intervention of three iron supplements

**DOI:** 10.1038/s41598-020-79971-y

**Published:** 2021-01-14

**Authors:** Qi Zhang, Xiao-Min Lu, Min Zhang, Chen-Ying Yang, Si-Yuan Lv, Shi-Fen Li, Cai-Yun Zhong, Shan-Shan Geng

**Affiliations:** 1 Department of Nutrition and Food Safety, Center for Global Health, School of Public Health, Nanjing Medical University, 101 Longmian Avenue, Jiangning District, Nanjing, 211166 China; 2 Center for Global Health, School of Public Health, Nanjing Medical University, Nanjing, 211166 China; 3 Safety Assessment and Research Center for Drug, Pesticide and Veterinary Drug of Jiangsu Province, Nanjing Medical University, Nanjing, 211166 China

**Keywords:** Nutrition, Paediatrics

## Abstract

Iron deficiency anemia (IDA) is a common micronutrient deficiency among pregnant women with severe consequences including impaired immuno-inflammatory system, premature birth, fetal death etc. The present study aimed to investigate the effects of three iron supplements on IDA female rats and their offspring. The IDA female rat model was established with low iron diet and the rats were then mated. After pregnancy, rats were fed diets containing different iron supplements (iron polysaccharide complex, iron protein succinylate and ferrous sulfate) until their offspring were 42 days old. Pregnancy outcomes, haematological, iron metabolism, physical and neurological development indexes were determined. The results showed that all three iron supplements improved the levels of hematological parameters of both mother and offspring rats. After iron supplementation, serum iron, transferrin saturation and serum ferritin levels were increased compared with the IDA group. The level of ferritin light chain in the liver and spleen of both mother and offspring rats in iron supplemented groups was significantly higher than that of the IDA group. The average number of born alive per litter in the iron treatment groups was significantly higher than that in the IDA group. Iron supplements also improved the physical growth and neurobehavioral development of offspring rats. It was also found that iron supplementation improved the expression of ferritin light chain and the synaptic growth associated proteins in the brain and hippocampus. No significant difference was found in the efficacy of three iron supplements. These results suggest that pregnant and postpartum IDA affects pregnancy outcomes, offspring physical development and causes neural impairment. Sufficient iron supplementation can significantly improve IDA and its adverse effects on both mother and offspring.

## Introduction

Iron is an essential micronutrient in human body and its deficiency leads to anemia along with a myriad of serious consequences^1^. Lack of adequate iron in diet or malabsorption will cause iron deficiency anemia (IDA), which affects millions of people throughout the world, especially among pregnant women. Because of the increased iron requirements during pregnancy, pregnant women are recognized as the group most vulnerable to IDA. Estimated by the World Health Organization (WHO), the prevalence of anemia in pregnant women is 38%^1^.

IDA during pregnancy can severely impair maternal and fetal outcomes. In the mother, IDA is associated with reduced physical performance, increased fatigue level, reduced cognitive performance, increased risk of infection and hospitalization, and inhibited lactation^2^. Also, pregnant women with anemia are at a greater risk of perinatal mortality and morbidity^3,4^. Adverse consequences for the fetus include spontaneous abortion, premature delivery, intrauterine fetal death, low birth weight, small for gestational-age babies, hypertension, neurologic impairment, etc.^5^.

Oral iron supplementation is an effective treatment for IDA during pregnancy^6^. The most frequently used oral iron preparations are ferrous sulfate (FS), ferrous fumarate, ferrous glycine sulfate, and ferrous gluconate. As early as 1998, the efficacy and tolerability of iron protein succinylate (IPS) in the treatment of iron deficiency in children were reported. Then, the use of succinic acid in the treatment of adults, pregnant women and premature IDA was reported^7–9^. In 2019, Córdova A et al. showed that supplementation with IPS improved haematological indexes in professional athletes^10^. However, the effects of IPS on pregnancy outcome and offspring physical and neural development have not been reported. Iron polysaccharide complex (IPC) is composed of low molecular weight polysaccharide and iron, in which the iron content is 46%. IPC does not contain free iron ions, so there is no corrosion and irritation to gastrointestinal mucosa caused by iron ions. Studies have shown that IPC can effectively treat IDA and improve haematological parameters^11–13^. In China, some studies reported the effects of IPC on IDA in pregnant women and its effects on pregnancy outcomes, but few studies have examined the growth and development of newborns after birth.

In this study, we initially established a rat model with IDA by using a combination of low iron diet with bloodletting and deionized water. IDA female rats were then allowed to proceed mating with males and conceiving. Next, in addition to determining the effects of IPS, IPC, and FS on pregnancy outcomes, we also examined the haematological and immuno-inflammatory indexes of the mother rats and offspring rats, as well as the physical and neural development of the offspring rats with those iron supplements.

## Results

### Establishment of female rat model with iron deficiency anemia

**Figure 1:**
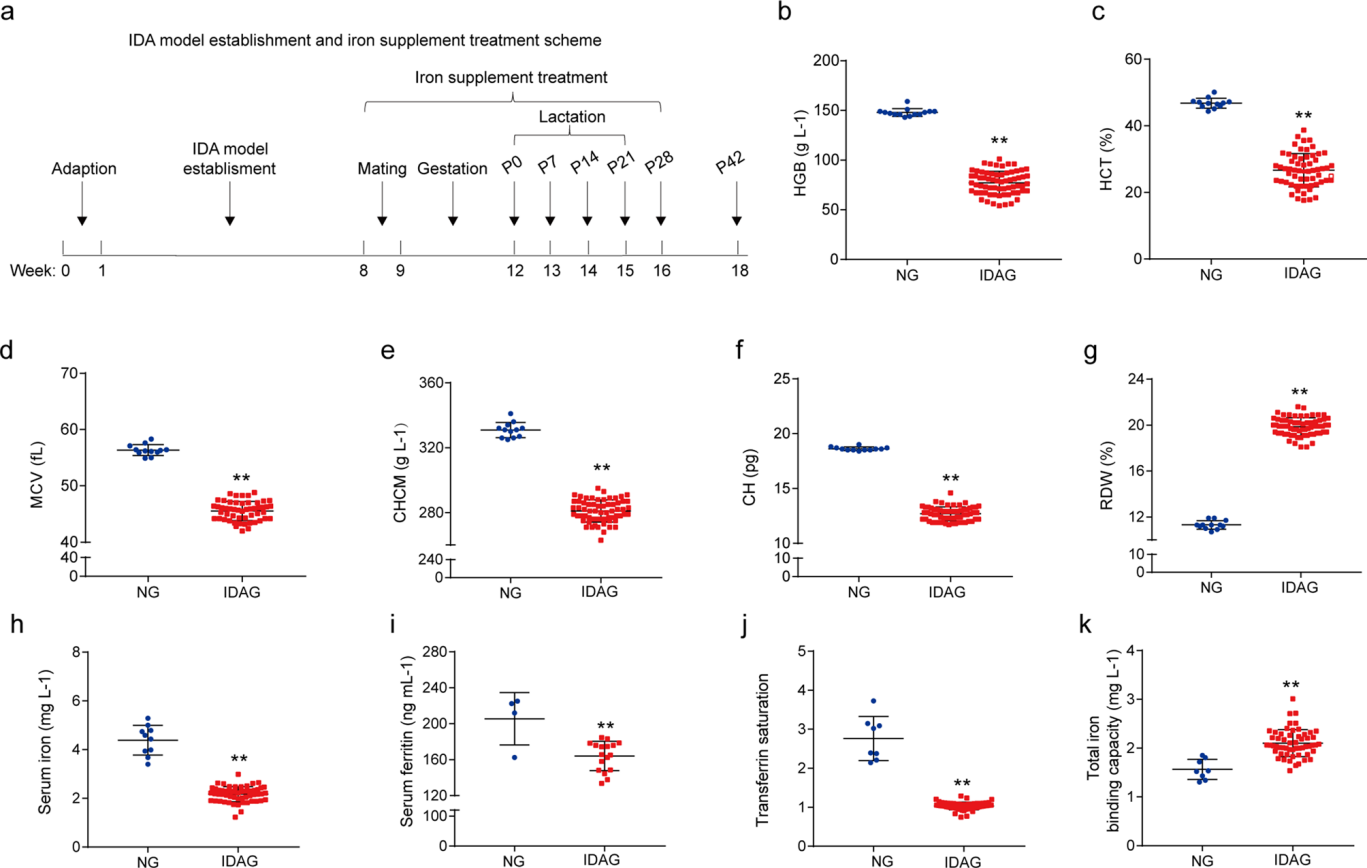
Establishment of iron deficiency anemia model in female rats. (**a**) Scheme of IDA model establishment and iron supplement treatment. The whole blood of maternal rats was collected and haematological indexes were tested. (**b**) HGB. (**c**) HCT. (**d**) MCV. (**e**) CHCM. **f** CH. (**g**) RDW. The serum of maternal rats was collected and tested. (**h**) SI. (**i**) SF. (**j**) TS. (**k**) TIBC. *HGB* haemoglobin, *HCT* Hematocrit, *MCV* mean corpuscular volume, *CHCM* cell haemoglobin concentration mean, *CH* haemoglobin content of red blood cell, *RDW* red cell volume distribution width, *SI* serum iron, *SF* serum ferritin, *TS* transferrin saturation, *TIBC* total iron binding capacity. Data are presented as mean ± SD (*n* = 12). **p < 0.01, compared with NG. Unpaired t-test was used for comparison between two groups.

IDA model establishment and iron supplement treatment scheme is shown in Fig. 1a. After 8 weeks of treatment with low iron diet plus deionized water and weekly bloodletting, the IDA model group (IDAG) rats had significantly lower levels of haemoglobin (HGB) (P < 0.001), hematocrit (HCT) (P < 0.001), mean corpuscular volume (MCV) (P < 0.001), cell haemoglobin concentration mean (CHCM) (P < 0.001), haemoglobin content of red blood cell (CH) (P < 0.001) and significantly higher red cell volume distribution width (RDW) (P < 0.001) than normal control group (NG) (Fig. 1b–g). Further analysis revealed that the levels of serum iron (SI), serum ferritin (SF) and transferrin saturation (TS) in IDA group were significantly lower than those in normal control group, and total iron binding capacity (TIBC) was significantly higher in IDA group (Fig. 1h–k).

### Effects of iron supplementation on maternal rats

#### Pregnancy outcomes

To determine whether maternal iron supplement could improve the pregnancy outcomes, the parturition rate, average number of total born per litter, and average number of total born alive per litter were recorded in anemia rats supplemented with or without iron during the entire gestation period. As shown in Table 1, the parturition rate of the IDA group was 44.83%, which was lower than that of NG (79.26%). In IDA group, the average number of total born per litter and average number of total born alive per litter were significantly lower than that in NG (Table 1). The average number of total born per litter in IPC high-dose group (IPC-H), IPS high-dose group (IPS-H), FS low-dose group (FS-L), FS high-dose group (FS-H), and the average number of total born alive per litter in IPS-L, IPS-H, FS-L, and FS-H groups were significantly higher than that in IDA group (Table 1). These results suggested that maternal iron supplementation significantly improved pregnancy outcomes.

**Table 1:** Pregnancy outcome of maternal rats.

	NG	IDAG	IPC-L	IPC-H	IPS-L	IPS-H	FS-L	FS-H
Parturition rate (%)	79.26	44.83	80	66.7	73.33	80.00	86.67	60.00
Average No. of total born per litter	7.89 ± 1.94	3.77 ± 2.00**	5.83 ± 2.25	6.40 ± 2.01^#^	6.09 ± 2.17	6.75 ± 3.05^#^	6.92 ± 1.60^##^	8.00 ± 2.45^##^
Average No. of total born alive per litter	7.78 ± 1.99	2.92 ± 1.44**	4.79 ± 2.83	4.50 ± 2.84	6.00 ± 2.10^#^	6.42 ± 2.94^##^	6.85 ± 1.63^##^	7.33 ± 3.61^##^

* *p* < 0.05, ** *p* < 0.01, compared with NG, # *p* < 0.05, ## *p* < 0.01, compared with IDAG. One-way ANOVA followed by Tukey multiple comparison test was used for comparison among 8 different groups.

#### Haematological and immune-inflammatory indexes of maternal rats

**Figure 2:**
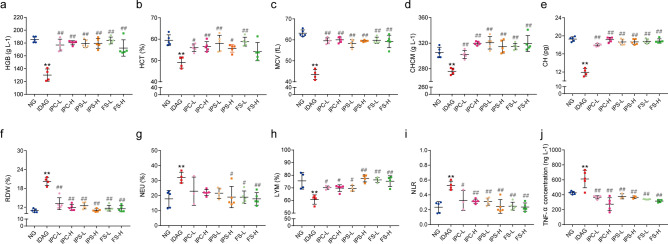
Haematological indexes of maternal rats after iron supplement treatment. The whole blood of maternal rats was collected and the haematological indexes were tested. (**a**) HGB. (**b**) HCT. (**c**) MCV. (**d**) CHCM. (**e**) CH. (**f**) RDW. (**g**) NEU. (**h**) LYM. (**i**) NLR. (**j**) TNF-α. *HGB* haemoglobin, *HCT* Hematocrit, *MCV* mean corpuscular volume, *CHCM* cell haemoglobin concentration mean, *CH* haemoglobin content of red blood cell, *RDW* red cell volume distribution width, *NEU* neutrophil, *LYM* lymphocyte, *NLR* neutrophil-to-lymphocyte ratio, *TNF-α* tumor necrosis factor α. Data are presented as mean ± SD (*n* = 5). *p < 0.05, **p < 0.01, compared with NG, ^#^*p* < 0.05, ^##^*p* < 0.01, compared with IDAG. One-way ANOVA followed by Tukey multiple comparison test was used for comparison among 8 different groups.

Figure 2 shows the haematological indexes measured in blood collected from different groups. The levels of HGB, HCT, MCV, CHCM and CH in the IDA group were significantly lower, and RDW was significantly higher than those in normal control group. After intervention with three iron supplements, HGB, HCT, MCV, CHCM, CH levels were significantly increased and RDW was significantly decreased (Fig. 2a–f).

As shown in Fig. 2g–j, the IDA group had significantly lower lymphocyte (LYM) level, and significantly higher neutrophil (NEU), neutrophil-to-lymphocyte ratio (NLR) and TNF-α levels than NG group. After intervention, the levels of NEU, NLR and TNF-α in the six iron supplemented groups were significantly lower than those in the IDA group (*p* < 0.05), while the level of LYM was significantly higher (*p* < 0.05). These results suggested that iron supplements improved immune-inflammatory status altered by IDA.

#### SI, TIBC, TS and SF levels

**Figure 3:**
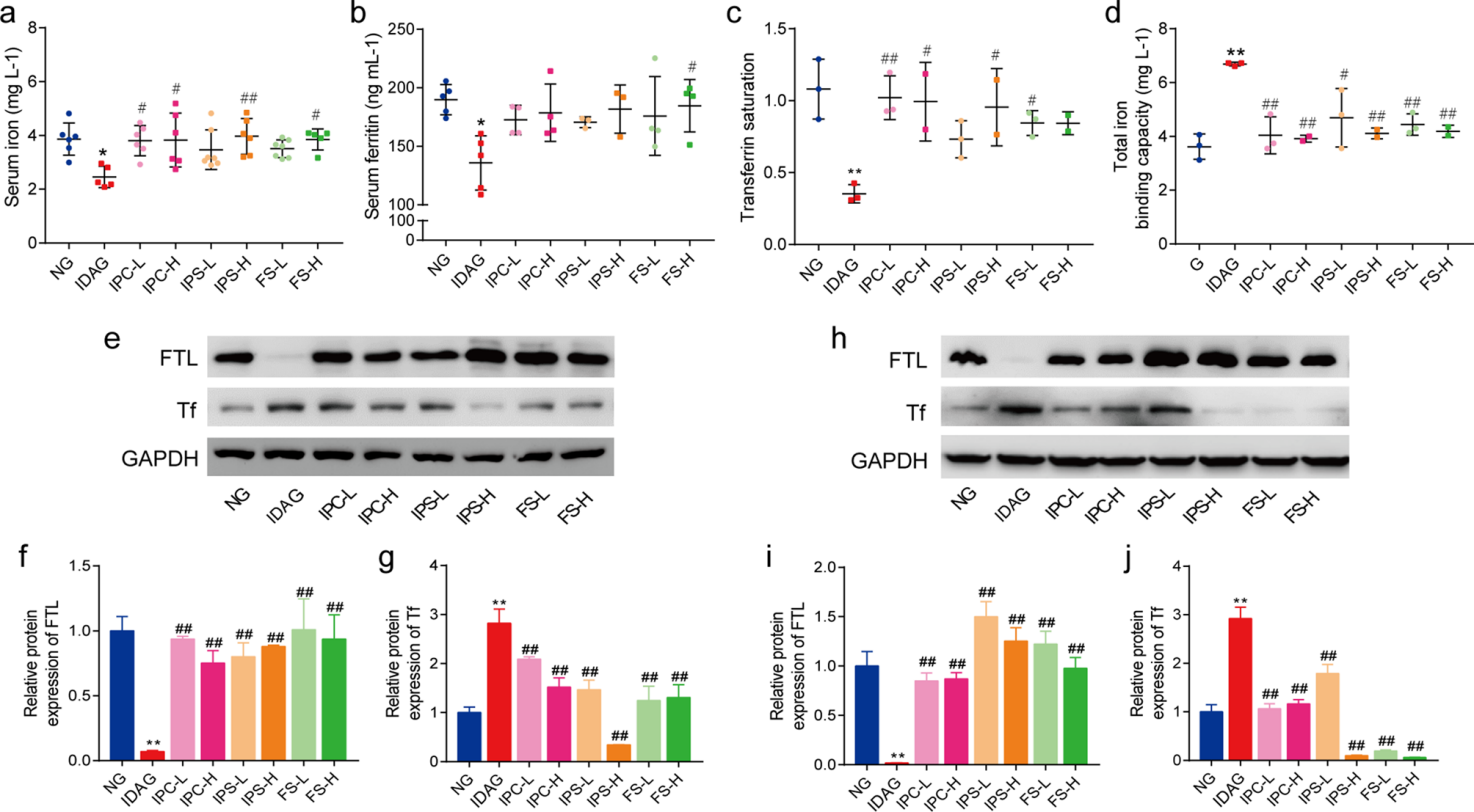
Iron related indexes of maternal rats after iron supplement treatment. The serum of maternal rats was collected and tested. (**a**) SI. (**b**) SF. (**c**) TS. (**d**) TIBC. Data are presented as mean ± SD (*n* = 6). Western blot analysis for FTL and Tf in liver (**e**–**g**) and spleen (**h**–**j**). The quantification of western blotting was provided in supplementary material. Data of Western blot analysis (mean ± SD) are expressed as the ratio of the relative contents between the value from IDA group and NG group and six iron treatment groups (n = 3). The relative contents of target proteins were quantified using the ratio between the optical density (OD) of target protein and the amount of the housekeeping protein GAPDH. *SI* serum iron, *SF* serum ferritin, *TS* transferrin saturation, *TIBC* total iron binding capacity, *FTL* ferritin light chain, *Tf* transferrin. *p < 0.05, **p < 0.01, compared with NG, ^#^*p* < 0.05, ^##^*p* < 0.01, compared with IDAG. One-way ANOVA followed by Tukey multiple comparison test was used for comparison among 8 different groups.

SI, TIBC, TS and SF levels are shown in Fig. 3a–d. At 28 days postpartum, the levels of SF, SI and TS in the IDA group were still significantly lower than those in NG (*p* < 0.05). In contrast, TIBC level in the IDA group was significantly higher (*p* < 0.05). After iron supplementation, the levels of SI, TS and SF in IPC, IPS, and FS groups were increased compared with the IDA group, while TIBC level in these iron treatment groups was significantly decreased.

#### Liver and spleen ferritin light chain (FTL) and transferrin (Tf) levels

Figure 3e–j shows iron accumulation and iron uptake in the liver and spleen of different groups. The level of FTL in the liver and spleen of rats in the IDA group was significantly lower than the NG group and the iron supplemented groups, while the level of Tf was significantly higher. These results suggested that iron accumulation in liver and spleen were reduced and iron transport capacity was increased in IDA group, and iron stores in the liver and spleen was increased after iron supplementation.

### Effects of iron supplementation on offspring rats

#### Haematological indexes of offspring

**Figure 4:**
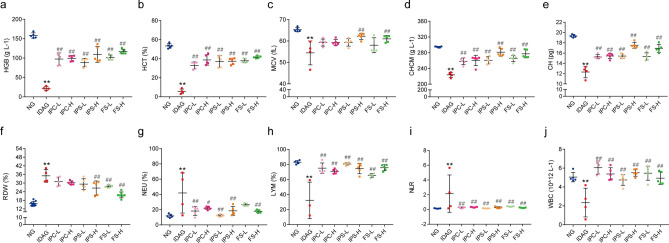
Haematological indexes of offspring rats after iron supplement treatment. The whole blood of offspring rats was collected and the haematological indexes were tested. (**a**) HGB. (**b**) HCT. (**c**) MCV. (**d**) CHCM. (**e**) CH. (**f**) RDW. (**g**) NEU. (**h**) LYM. (**i**) NLR. (**j**) WBC. *HGB* haemoglobin, *HCT* Hematocrit, *MCV* mean corpuscular volume, *CHCM* cell haemoglobin concentration mean, *CH* haemoglobin content of red blood cell, *RDW* red cell volume distribution width, *NEU* neutrophil, *LYM* lymphocyte, *NLR* neutrophil-to-lymphocyte ratio, *WBC* white blood cells count. Data are presented as mean ± SD (*n* = 5). *p < 0.05, **p < 0.01, compared with NG, ^#^*p* < 0.05, ^##^*p* < 0.01, compared with IDAG. One-way ANOVA followed by Tukey multiple comparison test was used for comparison among 8 different groups.

At 28 days after birth, the haematological parameters of the offspring were similar to those of the mother rats. The HGB, HCT, MCV, CHCM, and CH levels of the rats in IDA group were significantly lower than those in NG group, while RDW level was significantly higher. After iron supplementation, the levels of HGB, HCT, MCV, CHCM, and CH in the offspring rats were significantly higher than those in the IDA group, and RDW level was significantly lower (Fig. 4a–f).

#### Immune-inflammatory indexes of offspring

Similarly, increased NEU and NLR levels and decreased LYM and WBC levels in the offspring rats of the IDA group were observed. The levels of these indexes in iron supplemented groups were improved, which indicated that iron intervention improved the immune-inflammatory status in the offsprings of IDA rats (Fig. 4g–j).

#### FTL and Tf levels in liver and spleen of offspring

**Figure 5:**
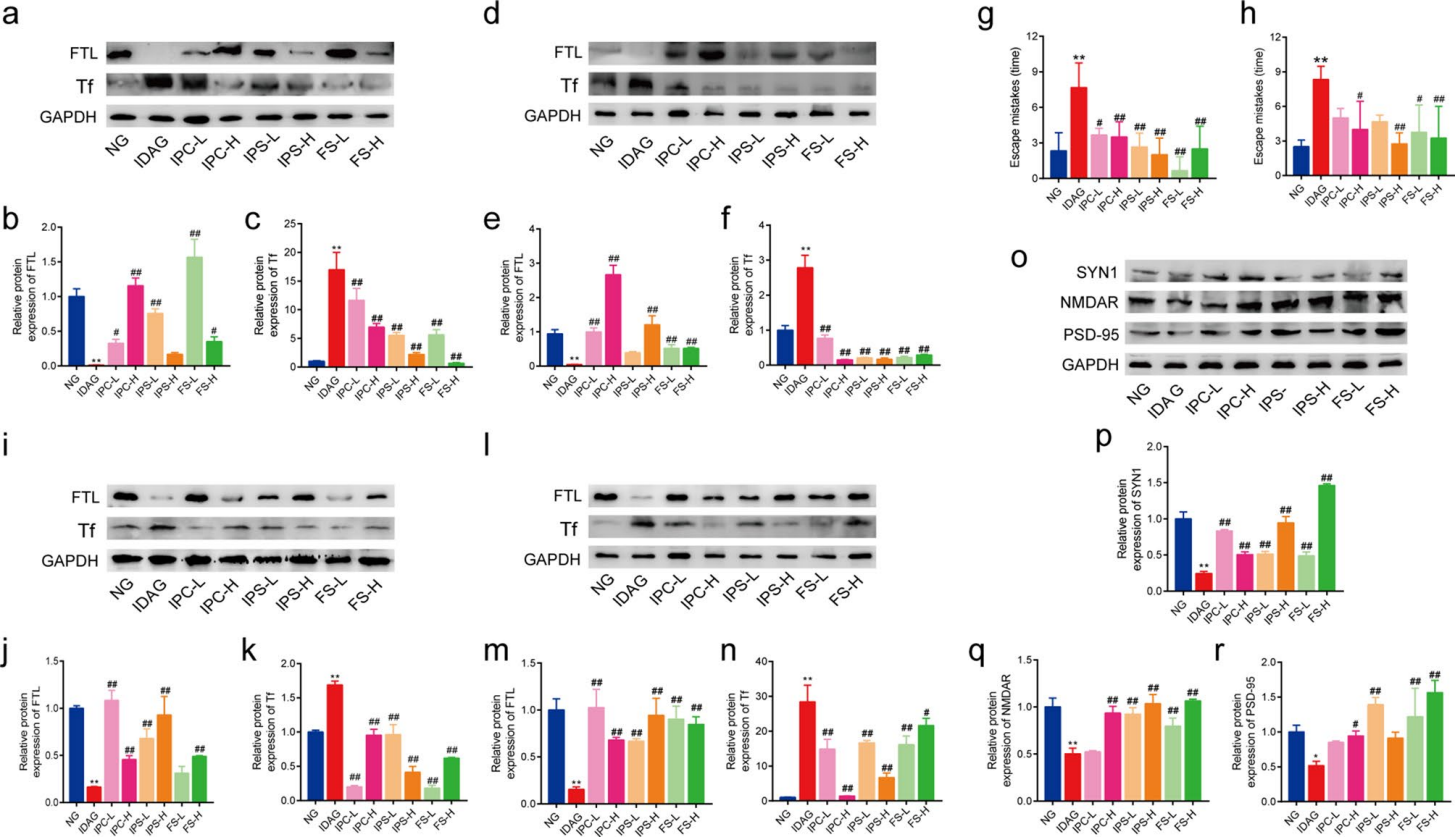
Iron related indexes and neural development of offspring rats after iron supplement treatment. Western blot analysis for FTL and Tf in liver (**a**–**c**) and spleen (**d**–**f**). Morris water maze test for day 1 escape latency (**g**) and day 2 escape latency (**h**). Western blot analysis for FTL and Tf in brain (**i**–**k**) and hippocampus (**l**–**n**), and for SYN1, NMDAR, PSD-95 in hippocampus (**o**–**r**). The quantification of western blotting was provided in supplementary material. *FTL* ferritin light chain, *Tf* transferrin, *SYN1* synapsin 1, *NMDAR* N-methyl-D-aspartate receptor, *PSD-95* postsynaptic density protein 95. Data of Western blot analysis (mean ± SD) are expressed as the ratio of the relative contents between the value from IDA group and NG group and six iron treatment groups (n = 3). The relative contents of target proteins were quantified using the ratio between the optical density (OD) of target protein and the amount of the housekeeping protein GAPDH. **p < 0.01, compared with NG, ^#^*p* < 0.05, ^##^*p* < 0.01, compared with IDAG. One-way ANOVA followed by Tukey multiple comparison test was used for comparison among 8 different groups.

As shown in Fig. 5a–c, the protein expression of FTL in the liver and spleen of the offspring rats in IDA group was significantly lower than NG group, which was reversed by iron supplement. The expression of Tf in the liver and spleen of IDA offspring rats was significantly higher than that of NG and iron supplemented groups (Fig. 5d–f). These results suggested that the offsprings of IDA rats were also in a state of iron deficiency, which indicated the effects of IDA during pregnancy and lactation.

#### Physical growth and development of offspring

At postnatal day (PND) 7, 14, 21 and 28, the offspring’s body weight of the IDA group was significantly lower than that of NG group and iron supplemented groups. The offspring of the IDA group gained only about 14 g of body weight in 21 days, while iron supplemented groups gained more than 50 g in 21 days (Table 2). The time of out of hair and tooth eruption in the IDA group was later than that in NG group and iron supplemented groups. At PND 28, the rate of born alive per litter in the IDA group was 23%, which was about 98% in NG and iron supplemented groups. These results indicated that the anemic dams not only delivered fewer but also lower survival rate (Table 3).

**Table 2:** The body weight of offspring rats.

Group	Average weight of pups born alive at PND7 (g)	Average weight of pups born alive at PND14 (g)	Average weight of pups born alive at PND21 (g)	Average weight of pups born alive at PND28 (g)
NG	15.61 ± 1.704	30.74 ± 3.896	47.52 ± 7.016	64.98 ± 9.381
IDAG	9.823 ± 2.012**	15.55 ± 3.947**	19.94 ± 5.614**	23.63 ± 3.217**
IPC-L	14.95 ± 2.524^##^	28.18 ± 5.102^##^	43.64 ± 6.821^##^	65.04 ± 8.777^##^
IPC-H	16.85 ± 4.691^##^	34.21 ± 6.12^##^	51.35 ± 8.356^##^	71.28 ± 13.25^##^
IPS-L	15.85 ± 3.656^##^	31.83 ± 5.042^##^	48.67 ± 6.861^##^	65.96 ± 10.55^##^
IPS-H	16.02 ± 2.135^##^	32.06 ± 3.813^##^	45.86 ± 7.129^##^	60.92 ± 11.95^##^
FS-L	16.47 ± 2.016^##^	33.09 ± 3.079^##^	49.98 ± 6.874^##^	70.41 ± 8.834^##^
FS-H	16.38 ± 2.072^##^	34.2 ± 5.055^##^	50.79 ± 6.595^##^	62.34 ± 11.47^##^

* *p* < 0.05, ** *p* < 0.01, compared with NG, # *p* < 0.05, ## *p* < 0.01, compared with IDAG. One-way ANOVA followed by Tukey multiple comparison test was used for comparison among 8 different groups.

**Table 3:** The development and PND 28 survival of offspring rats.

Group	Out of the hair (d)	Tooth eruption (d)	Eyes open (d)	Rate o born alive per litter at PND28 (%)
NG	3.313 ± 0.6021	7.176 ± 0.809	14.62 ± 0.5064	0.9851 ± 0.04342
IDAG	4.545 ± 1.128**	8.545 ± 1.508**	14.44 ± 0.7265	0.2303 ± 0.3959**
IPC-L	3.083 ± 0.5149^##^	6.917 ± 0.5149^##^	14.33 ± 0.7785	0.9861 ± 0.04811^##^
IPC-H	3.1 ± 0.7379^##^	6.6 ± 0.6992^##^	14.22 ± 1.394	0.9732 ± 0.05663^##^
IPS-L	3.6 ± 0.5164^#^	6.727 ± 0.9045^##^	14.44 ± 0.8819	0.9886 ± 0.03769^##^
IPS-H	3.091 ± 0.5394^##^	6.909 ± 0.8312^##^	14.36 ± 0.809	0.9467 ± 0.1069^##^
FS-L	3.308 ± 0.7511^##^	7.077 ± 0.8623^##^	14.25 ± 0.6216	0.9890 ± 0.03962^##^
FS-H	3 ± 0.5345^##^	7.375 ± 0.5175^#^	14 ± 0.8944	1.000 ± 0.0^##^

* *p* < 0.05, ** *p* < 0.01, compared with NG, # *p* < 0.05, ## *p* < 0.01, compared with IDAG. One-way ANOVA followed by Tukey multiple comparison test was used for comparison among 8 different groups.

#### Neural growth and development of offspring

The onset time of surface righting reflex, cliff avoidance, negative geotropism and air righting reflex was observed and recorded to evaluate the neural growth and development of the offspring. As shown in Table 4, the onset time of surface righting reflex, cliff avoidance, negative geotropism and air righting reflex in the IDA group was later than that in NG group. However, the onset time of these indexes in the iron supplemented groups was significantly earlier than that in the IDA group, and there was no significant difference with NG group.

**Table 4:** The nerve growth and development of offspring rats.

Group	Surface righting reflex (d)	Cliff avoidance reflex (d)	Negative geotropism (d)	Air righting reflex (d)
NG	3.526 ± 0.7723	4.684 ± 0.8201	4.632 ± 0.8951	10.75 ± 1.342
IDAG	5.273 ± 1.009**	6.909 ± 1.375^##^	6.818 ± 1.168^##^	12.67 ± 1.323**
IPC-L	4.083 ± 0.6686^#^	5.333 ± 0.7785^##^	5.25 ± 0.7538^##^	10.67 ± 1.303^#^
IPC-H	3.6 ± 0.6992^##^	4.5 ± 0.7071^##^	4.5 ± 0.7071^##^	10.2 ± 1.476^##^
IPS-L	3.545 ± 0.8202^##^	4.364 ± 0.809^##^	4.364 ± 0.809^##^	10.64 ± 1.027^#^
IPS-H	3.455 ± 0.5222^##^	4.273 ± 0.6467^##^	4.364 ± 0.6742^##^	9.818 ± 1.834^##^
FS-L	3.538 ± 1.506^##^	4.692 ± 0.6304^##^	4.615 ± 0.5064^##^	10.6 ± 1.506^#^
FS-H	3.125 ± 0.3536^##^	4.5 ± 0.5345^##^	4.5 ± 0.5345^##^	10.63 ± 1.598^#^

* *p* < 0.05, ** *p* < 0.01, compared with NG, # *p* < 0.05, ## *p* < 0.01, compared with IDAG. One-way ANOVA followed by Tukey multiple comparison test was used for comparison among 8 different groups.

#### Morris water maze test

On day 1 of the probe trial, the offspring rats of the IDA group showed increased escape mistakes compared to NG group, indicating impaired spatial learning ability. Compared with the IDA group, iron intervention groups showed a significant decrease in escape mistakes (Fig. 5g). On day 2 of the probe trial, the escape mistakes of the rats in the IDA group were still more than that in NG group and iron intervention groups (Fig. 5h). These results indicated that IDA affected spatial learning ability in offspring and this adverse effect was improved by iron supplementation.

#### Expression of iron related proteins and synaptic growth associated proteins in brain and hippocampus

We examined the expression of iron related proteins, such as FTL and Tf, in the brain and hippocampus of offspring rats. The level of FTL was significantly lower in IDA group than that of other groups, and the level of Tf was oppositely altered (Fig. 5i–n). Then we detected the synaptic growth associated proteins in hippocampus. As expected, the levels of SYN1, NMDAR and PSD-95 were significantly decreased in IDA rats (Fig. 5o–r), while the expression of these proteins was increased in iron intervention groups. Taken together, the IDA offspring rats showed abnormal and retarded neurodevelopment, which was improved by iron supplement.

## Discussion

IDA during pregnancy is a common health problem, even in high-income countries. Iron is an essential micronutrient which is important not only for carrying oxygen but also for the catalytic activity of various enzymes. Therefore, iron deficiency anemia during pregnancy has both short term and long term effects on the pregnant woman, the puerperal woman, the fetus and the infant^14–18^. In the present study, based on the establishment of IDA female rat model, the adverse effects of IDA on mother and offspring as well as the improvement effects of iron supplementation were observed.

In this study, the IDA model was established in a period of 8 weeks with low iron diet (9 ppm), deionized drinking water and bleeding once a week. HGB is the most commonly used indicator of iron deficiency anemia. In our experiments, HGB < 90 g/L was used as the successful criterion of the IDA model. Our study showed that this method had about a rate of 73% IDA (HGB < 90 g/L) at eight weeks. In addition, some other indicators such as HCT, MCV, CHCM, CH, and RDW also showed significant changes. Serum iron, serum ferritin, TS, and TIBC are also important indicators reflecting the iron metabolism in vivo. There were also significant alterations in these indicators in our IDA animal model.

The liver and spleen are the principal iron storage organs and critical for the regulation of systemic iron homeostasis. Hepatic iron deposition is properly controlled by the liver iron uptake and export system. Hepatic iron uptake depends on Tf, transferrin receptor 1/2, et al.^18,19^. The excess iron is stored as ferritin which consists of 24 subunits of heavy (FTH) or light (FTL) isoforms in a spherical shell that plays a central role in the intracellular iron balance^20–22^. In this study, Tf protein expression level was up-regulated and FTL was down-regulated in the liver and spleen of IDA rats. These results indicated that iron stores in the liver and spleen decreased during pregnancy and lactation up to 14 weeks.

Iron deficiency affects performance during pregnancy and delivery, lactation performance, and immuno-inflammatory status^2^. Severe anemia can also increase perinatal maternal mortality. In this study, although no maternal death was observed in the IDA group, the abnormality of immuno-inflammatory parameters was observed. Serum neutrophils, lymphocytes and TNF-α levels are common markers of immuno-inflammatory response. NLR is a simple parameter that makes it easy to assess the inflammatory state of a subject. It has been shown to be a useful predictor and marker of inflammation, infection, postoperative complications, etc^23–25^. In this study, the levels of neutrophils, NLR and TNF-α in the IDA group were significantly higher than those in control group and iron supplemented groups, along with significant lower level of LYM. These data suggested that IDA impaired the immune-inflammatory status. As a key nutrient for the developing fetus, neonate, infant, and child, the demand of iron is high during the early stages of life because it is critical for the production of new red blood cells and muscle cells as well as brain development. A recent study of anemia and iron deficiency in pregnancy in Southern India showed that IDA in pregnancy was associated with higher risk of low birth weight, preterm birth, gestational age at birth and infant WAZ scores^26^. Our study also showed that the number of live births per litter in IDA group was significantly lower than that in the normal control group, and the body weight and 28-day weight gain were significantly lower than those in other groups with sufficient iron. The time of tooth eruption, eye opening and hair growth of the rats in the IDA group was also later than that in the normal control group and iron supplemented groups. The 28-day survival rate in IDA group was only 23%, in comparison to about 98% in NG and iron supplemented groups. These results suggested that maternal iron deficiency and anemia during pregnancy affected the growth and development of offspring.

In early embryonic life, iron is already necessary for normal brain development due to the proliferation, migration, and differentiation of neural progenitor cells. Animal models of prenatal iron deficiency show abnormalities in brain structure, neurotransmitter system and myelin formation, resulting in acute brain dysfunction during the period of deficiency and persistence of various postnatal neurobehavioural abnormalities^27^. Studies of fully developed infants have shown that iron deficiency experienced during development can have chronic and irreversible damage to cognitive, memory and motor skills, indicating widespread effects of iron deficiency early in life on neurodevelopment. Our results illustrated that the IDA group had a lower level of neurodevelopment than iron supplemented groups. Synaptic growth and development proteins, including SYN1, NMDAR and PSD-95, were severely decreased in IDA rats. These alterations in turn retarded the neurobehavioral development, such as surface righting reflex, cliff avoidance, negative geotropism and air righting reflex, and spatial learning and memory ability.

Iron protein succinylate, iron polysaccharide complex and ferrous sulfate are all iron derivatives for the oral treatment of IDA. However, there was no study to determine the effects of these three iron supplements on pregnancy outcomes and offspring growth. Cancelo-Hidalgo et al. reported the incidence of overall adverse reactions was 7.3% for iron protein succinylate and 32.3% for ferrous sulfate, indicating that iron protein succinylate had a lower incidence of adverse effects than ferrous sulfate^28^. Since iron polysaccharide complex does not contain free iron ions, it causes little corrosion and irritation to gastrointestinal mucosa. In an intervention trial for anemia of prematurity, no notable adverse events were observed in either iron protein succinylate or iron polysaccharide complex group^7^. In the present study, we did not observe significant differences in the improvement effects of these three iron supplements. In the cases of severe iron deficiency anemia, iron supplementation can significantly improve maternal and offspring outcomes. Therefore, iron supplementation or not is more important for maternal and offspring than the type of iron supplement.

In conclusion, the effects of iron deficiency anemia on pregnant females were severe, leading to premature birth, miscarriage, significant reduction in the number of litters per litter and birth weight, as well as abnormalities in maternal immune-inflammatory status. It also had significant effects on the growth and development of offspring, both physically and neurologically. Therefore, when pregnancy anemia is diagnosed, iron supplements should be given to prevent and correct the adverse effects. Iron polysaccharide complex, iron protein succinylate and ferrous sulfate could significantly improve pregnancy outcomes and iron nutrition status in maternal IDA rats, and are beneficial for the growth and development of offspring. Therefore, they can be used as effective iron supplements for pregnant women.

## Materials and methods

### Materials

Iron protein succinylate (IPS) (5.46% iron content) was purchased from Raw Material Medicine Reagent Co., LTD (Nanjing, Jiangsu, China). Iron polysaccharide complex (IPC) (46% iron content) was purchased from Shanghai Pharmaceutical Group, Qingdao Growful Pharmaceutical Co., Ltd. (Qingdao, Shandong, China). Ferrous sulfate (FS) was purchased from Sigma Aldrich (St. Louis, MO, USA). Primary antibodies against transferrin (Tf), ferritin light chain (FTL) were obtained from Wuhan Sanying Biology Technology Co., Ltd (Wuhan, Hubei, China). Primary antibodies against Synapsin 1 (SYN1), N-methyl-D-aspartate receptor (NMDAR) and postsynaptic density protein 95 **(**PSD-95) were obtained from Affinity (OH, USA). The primary antibody for GAPDH was from Biogot Technology Co., Ltd. (Nanjing, Jiangsu, China). TNF-α ELISA Kit was purchased from SenBeiJia Biological Technology Co., Ltd (Nanjing, Jiangsu, China). Serum iron (SI) test kit, total iron binding capacity (TIBC) test kit, and serum ferritin (SF) test kit were purchased from Nanjing Jiancheng Bioengineering Inst (Nanjing, Jiangsu, China).

### Animals

All animals used in the study were purchased from Shanghai SLAC Laboratory Animal Co.,Ltd [Licence No: SCXK (Hu)2017-0005]. Five-week-old, female and male Wistar rats were housed in pathogen free environment in the animal house facility of Nanjing Medical University at regulated temperature 22 to 26 °C and under 12 h/12 h light/dark cycles. Free access to laboratory diet and drinking water was provided to the animals. Protocol adopted in the study was approved by the Institutional Animal Care and Use Committee (IACUC) of Nanjing Medical University. The animal study protocol approval number was IACUC-1812017. All experiments were performed in accordance with the approved guidelines and regulations by IACUC of Nanjing Medical University.

### Iron deficiency anemia model and treatment

Female rats were randomly divided into normal control group (NG) and iron deficiency anemia model group. Normal control animals were fed with normal laboratory diet containing 50 ppm iron during the experimental period. The animals in the IDA group were fed with low iron diet containing 9 ppm iron (Jiangsu Xie Tong Pharmaceutical Bio-engineering Co., Ltd., China) and deionized water ad libitum and followed by bleeding once a week for 8 weeks.

After 8 weeks, orbital blood was collected and haemoglobin (HGB) content was measured using an automatic biochemistry analyzer (ADVIA 2120i, Siemens, Germany). Rats with haemoglobin content below 90 g/L were considered to be IDA and were used in subsequent experiments. Except NG rats, the anemia animals were randomly divided into seven group: IDA model group (IDAG), low dose of iron polysaccharide complex group at iron dose of 30 mg·kg^−1^ (IPC-L), high dose of iron polysaccharide complex at iron dose of 50 mg·kg^−1^ (IPC-H), low dose of iron protein succinylate group at iron dose of 30 mg·kg^−1^ (IPS-L), high dose of iron protein succinylate at iron dose of 50 mg·kg^−1^ (IPS-H), low dose of ferrous sulfate at iron dose of 30 mg·kg^−1^ (FS-L), high dose of ferrous sulfate at iron dose of 50 mg·kg^−1^ (FS-H).

After grouping, female rats from each group and male rats were mated by 3:1. Mating was confirmed by detection of a vaginal plug at 8:00 in the morning. After mating, female rats were feed separately. NG rats were fed with normal diet. IDAG were fed with low-iron diet. Six treatment groups were fed with iron-containing diet (30 mg·kg^−1^ and 50 mg·kg^−1^) respectively. All groups of mother rats were fed until the 28th day after the birth of their offspring.

After birth, the number of live offspring per litter was recorded. The weight of the offspring was recorded weekly for 28 days after birth. The growth and development indexes, such as tooth eruption, eye opening and villus growth, were observed and recorded. The nerve reflex and motor coordination function indexes, such as the time of surface righting reflex, cliff avoidance, negative geotropism, and aerial righting reflex were tested and recorded. On the 28th day, the number of the surviving offspring was recorded and the survival rate was calculated. Most of the offspring in each group were sacrificed and blood and tissues were collected. Six rats in each group were fed to 42 days after birth and their learning ability was measured by Morris water maze test.

### Sample collection

On the 28th day, blood of the mother rats and the offspring rats was collected. The blood samples were collected in non-heparinized/heparinized microcapillary tubes from the rats’ retro-orbital plexus. A small amount of blood from each rat was immediately added to the automated hematology analyzer for determination. The remaining blood samples were centrifuged to obtain serum and were frozen at − 20 °C for further analysis. Next, all mother and offspring rats were sacrificed and the liver, spleen, brain and hippocampus were removed, rinsed with phosphate-buffered saline, weighed, and stored at − 80 °C for further analysis.

### Morris water maze test

The Morris water maze was a black rectangular flume, in which several diaphragms formed the maze, and one corner of the maze had a group of steps as the end point. Before the experiment, the maze was filled with water, with water depth of 10 cm and water temperature of 25 °C. On the first day of training, rats were placed on the step for 10 s to make them understand the existence of this safe area, rats were then placed at the starting point to let them swim freely, and the mistakes of climbing the safe step within 2 min were recorded. On the next day, the training of the first day was repeated again, and the mistakes of reaching the end point were recorded and the learning ability of the offspring rats was evaluated.

### Haematological analysis

Haemoglobin (HGB), hematocrit (HCT), mean corpuscular volume (MCV), red cell volume distribution width (RDW), cell haemoglobin concentration mean (CHCM), haemoglobin content of red blood cell (CH), lymphocyte (LYM), neutrophil (NEU) and white blood cells count (WBC) levels were measured using an automated hematology analyzer (ADVIA 2120i, Siemens, Germany).

### SI, TIBC and SF levels

Serum iron (SI) concentration and total iron binding capacity (TIBC) were measured using SI test kit and TIBC test kit (Nanjing Jiancheng Bioengineering Inst., Nanjing, Jiangsu, China) respectively according to the manufacturer's instructions. Transferrin saturation (TS) was calculated from SI to TIBC ratio as follows: TS (%) = [SI (mg/L)/TIBC (mg/L)] × 100%. Serum ferritin (SF) level was measured using double-antibody sandwich enzyme-linked immunosorbent assay (ELISA) kit according to the manufacturer's instructions (SenBeiJia Biological Technology Co., Ltd, Nanjing, Jiangsu, China).

### Inflammatory cytokine

The level of tumor necrosis factor α (TNF‐α) in rat serum was measured by ELISA kits according the manufacturer's instructions (SenBeiJia Biological Technology Co., Ltd, Nanjing, Jiangsu, China).

### Western blotting

Total proteins were extracted from the liver, spleen, brain and hippocampus tissues using RIPA buffer (Beyotime, Shanghai, China). Lysates from each sample were run on gels, electrotransferred onto polyvinylidene difluoride (Millipore, Billerica, MA, USA), and immunoblotted with primary antibodies. Horseradish peroxidase (HRP)-conjugated goat anti-rabbit IgG (ZSGB‐BIO, China) was used as secondary antibody. Immunoreactive proteins were visualized by enhanced chemiluminescence (Cell Signaling Technology, USA). Quantification of the immunoreactive bands was performed by using Image-Pro Plus 5.0.1.9 software (Media Cybernetics inc., Rockville, MD, USA).

### Statistical analysis

Data are expressed as means ± standard deviation (SD). Unpaired t-test and one-way ANOVA analyses were performed to compare the difference between two or multiple groups using GraphPad Prism 7.0 software. A *p* value of < 0.05 was considered statistically significant.

### Consent for publication

Consent for publication was obtained from all authors.

## Supplementary Information

Supplementary Information

## Data Availability

All data generated or analyzed during this study are included in this published article.
