# Supplementary material for: Adverse effects of iron deficiency anemia on pregnancy outcome and offspring development and intervention of three iron supplements
Source: Sci Rep. 2021 Jan 14;11:1347. doi: 10.1038/s41598-020-79971-y (PMC7809104; doi:10.1038/s41598-020-79971-y)

Adverse effects of iron deficiency anemia on pregnancy outcome and offspring development and intervention of three iron supplements

Qi Zhang [1], Xiao-Min Lu[1], Min Zhang[1], Chen-Ying Yang[1], Si-Yuan Lv[1], Shi-Fen Li[3], Cai-Yun Zhong [1,2*], Shan-Shan Geng [1,2*]

Affiliations

[1]Department of Nutrition and Food Safety, School of Public Health, Nanjing Medical University, Nanjing 211166, China

[2]Center for Global Health, School of Public Health, Nanjing Medical University, Nanjing 211166, China.

[3]Safety Assessment and Research Center for Drug, Pesticide and Veterinary Drug of Jiangsu Province, Nanjing Medical University, Nanjing, 211166, China.

*** Correspondence to:** Caiyun Zhong, M.D., Ph.D., DABT, Department of Nutrition and Food Safety, Center for Global Health, School of Public Health, Nanjing Medical University, 101 Longmian Avenue, Jiangning District, Nanjing, 211166, China. E-mail: cyzhong@njmu.edu.cn. Telephone: +86 25 86868450. Fax: +86 25 86868409; Shan-Shan Geng, Ph.D., Department of Nutrition and Food Safety, School of Public Health, Nanjing Medical University, 101 Longmian Avenue, Jiangning District, Nanjing, 211166, China. E-mail: gss9814@njmu.edu.cn. Telephone: +86 2586868450. Fax: +86 25 86868409.

Qi Zhang, Xiao-Min Lu and Min Zhang contributed equally to this work.

Supplementary Figure Original western blot membranes for membrane strips presented in Figure 4 and Figure 6 of the manuscript.

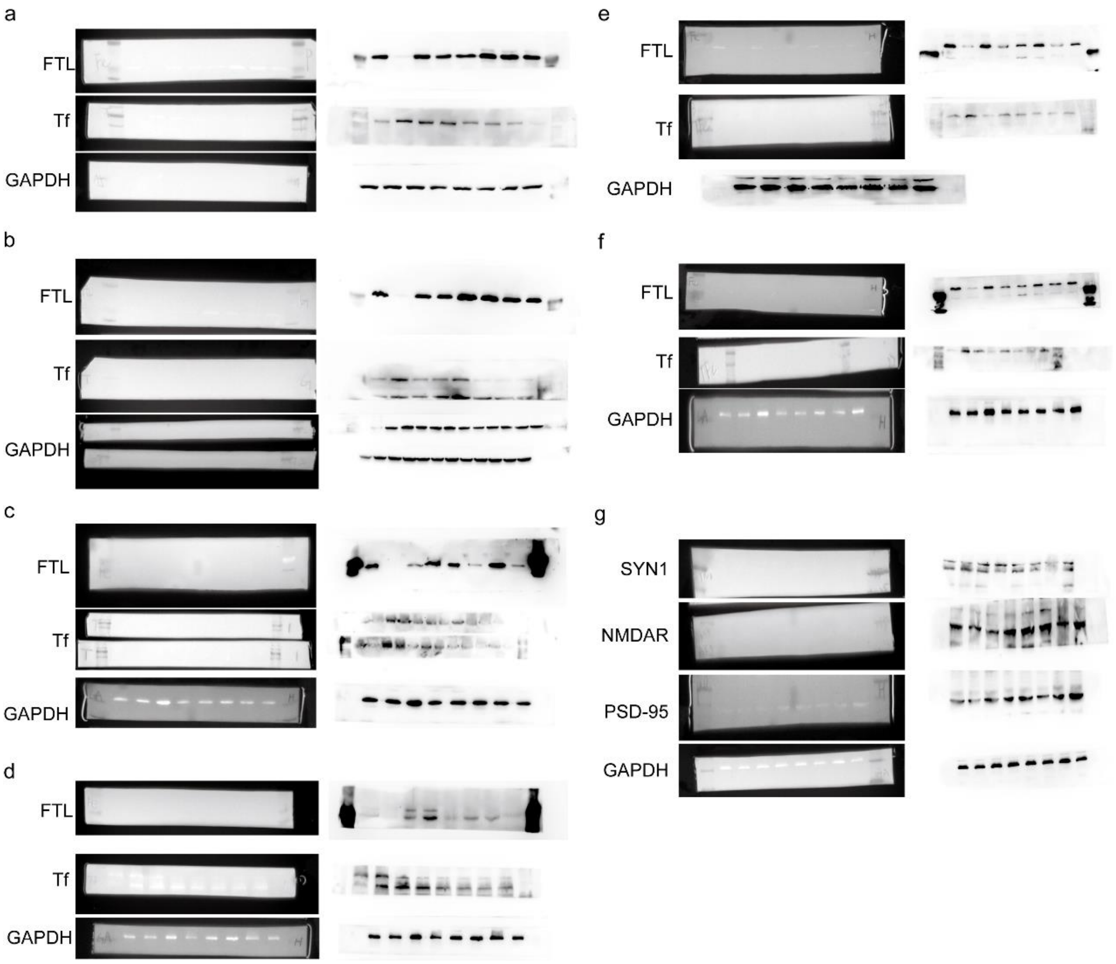

Supplement: Supplementary file 1 — Supplementary Information [file 41598_2020_79971_MOESM1_ESM.pdf]
